# Beneficial effects of varicocele embolization on semen parameters

**DOI:** 10.1186/2051-4190-24-9

**Published:** 2014-05-16

**Authors:** Julie Prasivoravong, François Marcelli, Laurent Lemaître, Pascal Pigny, Nassima Ramdane, Marie-Claire Peers, Valérie Mitchell, Jean-Marc Rigot

**Affiliations:** Department of Andrology, Lille University Hospital, Lille Cedex, France; Department of Radiology, Lille University Hospital, Lille Cedex, France; Department of Biochemistry and Molecular Biology, Lille University Hospital, Lille Cedex, France; Department of Biostatistics, Lille University Hospital, Lille Cedex, France; Biology of Reproduction Unit, Lille University Hospital, Lille Cedex, France; EA4308 Gametogenesis and Gamete Quality, University of Lille, Lille Cedex, France; Department of Andrology, CHRU Lille, Hôpital Calmette, Boulevard du Professeur Leclercq, 59037 Lille Cedex, France

**Keywords:** Varicocele, Sperm parameters, FSH, Inhibin B, Embolization, Varicocèle, Paramètres spermatiques, FSH, Inhibine B, Embolisation

## Abstract

**Background:**

The value of varicocele repair and the latter's impact on semen parameters are still subject to debate.

**Methods:**

We analyse changes over time in initially abnormal sperm parameters and serum concentrations of testosterone, FSH and inhibin B after embolization treatment of males with high-grade varicocele. From 2007 to 2012, we recruited 47 male infertile patients with clinically visible left varicocele in the resting patient and at least one abnormal semen parameter. Sperm parameters and serum levels of total testosterone, FSH and inhibin B were measured prior to retrograde embolization (M0) and then 3 (M3) and 6 (M6) months afterwards.

**Results:**

At M0, the median sperm concentration was 5.78 [0.84-37.70] × 10^6^/ejaculate. The mean ± SD sperm progressive motility, vitality and percentage of normal sperm were respectively, 21.83 ± 16.48%, 61.88 ± 15.98% and 12.88 ± 7.15%. The corresponding values at M3 were significantly higher (38.75 [3.96-95] × 10^6^/ejaculate, 29.32 ± 14.21%, 69.14 ± 14.86% and 19.03 ± 11.02%, respectively). The mean percentage of spermatozoa with a thin head was significantly lower at M6 (6.35 ± 5.29%) than at M0 (14.03 ± 13.09%). The mean serum testosterone, FSH and inhibin B levels did not change significantly over time.

**Conclusions:**

Embolization treatment in men with clinically visible left varicocele, abnormal sperm parameters and documented infertility is associated with a significant improvement in semen parameters including sperm head morphology.

## Résumé

**Introduction:**

L’intérêt du traitement de la varicocèle clinique et son impact sur les paramètres spermatiques sont de nos jours toujours débattus.

**Méthodes:**

Nous avons analysé l’évolution dans le temps des paramètres spermatiques et les concentrations sériques de testostérone, FSH et inhibine B d’hommes infertiles ayant une varicocèle de haut grade traitée par embolisation rétrograde. De 2007 à 2012, 47 patients ayant une varicocèle clinique de grade 3 et au moins un paramètre de sperme anormal ont été recrutés. Les paramètres spermatiques et les taux de testostérone totale, de FSH et d'inhibine B sérique ont été mesurés avant embolisation (M0) et 3 (M3) et 6 (M6) mois après embolisation.

**Résultats:**

La numération spermatique médiane initiale de la population était de 5,78 [0,84-37,70] × 10^6^ /éjaculat. Les moyennes ± déviation standard de la mobilité progressive, la vitalité et le pourcentage de spermatozoïdes normaux étaient respectivement de 21,83 ± 16,48%, 61,88 ± 15,98% et 12,88 ± 7,15%. A M3, les valeurs étaient significativement plus élevées (38,75 [3,96-95] × 10^6^/éjaculat, 29,32 ± 14,21%, 69,14 ± 14,86% et 19,03 ± 11,02% respectivement). Le pourcentage moyen de spermatozoïdes à tête amincie était significativement plus faible à M6 (6,35 ± 5,29%) qu’à M0 (14,03 ± 13,09%). Les taux sériques moyens de la testostérone, FSH et inhibine B étaient comparables tout au long de la prise en charge des patients.

**Conclusion:**

L'embolisation de la varicocèle de haut grade chez les hommes ayant au moins un paramètre spermatique anormal est associée à une amélioration significative des paramètres spermatiques et notamment des spermatozoïdes à tête amincie.

## Background

Varicocele is characterized by abnormal tortuosity and dilatation of the veins of the pampiniform plexus within the spermatic cord. The frequency of varicocele can be as high as 22% in the general population and 15% in adolescents [1]. Forty per cent of men with an abnormal semen analysis have varicocele [1]. In 1955, Tulloch reported on a link between varicocele repair and male fertility for the first time [2]. The relevance of varicocele repair in assisted reproductive technologies is still subject to debate because of the divergence in research findings [3–5]. The choice of the technique for varicocele repair and the procedure's impact on semen quality parameters are also much debated. An optimal treatment technique would be associated with the lowest possible recurrence and complication rates. Semen analysis is widely used as a marker of spermatogenesis. Despite heterogeneity in patient characteristics, diagnostic criteria, treatment methods and treatment outcomes, most researchers have found that varicocele repair is associated with a significant improvement in the sperm concentration (in 15 out of 22 studies) and progressive motility (in 12 out of 17 studies) [4]. Varicocele repair's effect on sperm morphology is less clear [6–8] and it has been suggested that significant morphological changes in germ cells can only be observed over long follow-up periods [7]. Menkveld et al. [9] showed that the measurement or evaluation of sperm morphology remains therefore a very important tool in the diagnosis of a male’s fertility potential and in the clinical decision making for the treatment of patients with infertility problems. Varicocele also affects hormonal status. Serum FSH and inhibin B levels are widely accepted markers of a patient's spermatogenic status. However, there is controversy over the nature and significance of serum hormone levels (and particularly inhibin B levels) after varicocele repair [10–12].

The aim of the present study was to analyse changes over time in initially abnormal sperm parameters (concentration, motility, vitality and morphology) and serum concentrations of testosterone, FSH and inhibin B after embolization treatment in males with high-grade varicocele.

## Methods

### Inclusion criteria

Male outpatients were recruited by the Department of Andrology at Lille University Hospital between March 2007 and June 2012, according to the French Urology Society's criteria for varicocele and male infertility [1]: documented infertility for more than 1 year, clinically visible left varicocele in the resting patient (i.e. grade III [1]) and at least one abnormal semen parameter in the initial semen analysis (according to the 2010 WHO criteria) [13]: a sperm concentration <39 × 10^6^/ejaculate and a percentage of progressive motility a + b <32%). Sperm morphology was assessed according to David’s modified classification [14] and the lower reference value for normal sperm was set to 15%.

### Scrotal ultrasonography

Varicocele was confirmed by scrotal ultrasonography in standing and supine patients and during the Valsalva manoeuvre. Varicocele was defined as a maximum vein diameter >3 mm and vein reflux > 2 seconds. The testicular volume (defined as 0.71 × length × width × height) was measured with an iStyle SSA 790A ultrasound system (Toshiba, Tochigi, Japan). The normal total (right plus left) testicular volume was defined as 32 mL or more [15].

### Hormonal status

Serum levels of total testosterone, FSH and inhibin B were assayed in blood samples drawn in the morning (8 to 10 AM) before embolization (M0) and then 3 (M3) and 6 (M6) months thereafter. Serum testosterone was measured in a radioimmunoassay (Coat-A-Count, Siemens, Los Angeles, CA, USA). The reference range was taken as 2.30–6.0 ng/mL [16]. Serum FSH levels were determined in an immunoassay (Abbott, Longford, Republic of Ireland) with a limit of detection of 0.05 IU/L and intra and interassay coefficients of variation below 3%. The reference range was 1.23-7.89 IU/L [17]. Serum inhibin B levels were measured using the two-site OBI Inhibin B enzyme immunoassay (Oxford Bio-Innovation Ltd, Oxford, UK). To improve the measurement range, an additional reference point (7.8 pg/mL) was added to the standard curve [16]. The mean normal serum inhibin B concentration was taken as 105–439 pg/mL [17].

### Semen samples

Sperm analyses were performed in the Biology of Reproduction Unit at Lille University Hospital. Samples were obtained by masturbation after 2–5 days of sexual abstinence. After liquefaction, standard semen parameters (volume, concentration, motility and vitality, i.e. the percentage of live spermatozoa) were measured according to the 2010 WHO guidelines [13]. Semen analyses were performed twice prior to embolization (M0) (to confirm the semen parameters) and then 3 and 6 months afterwards (M3 and M6, respectively).

### Intervention: left embolization

**Figure 1:**
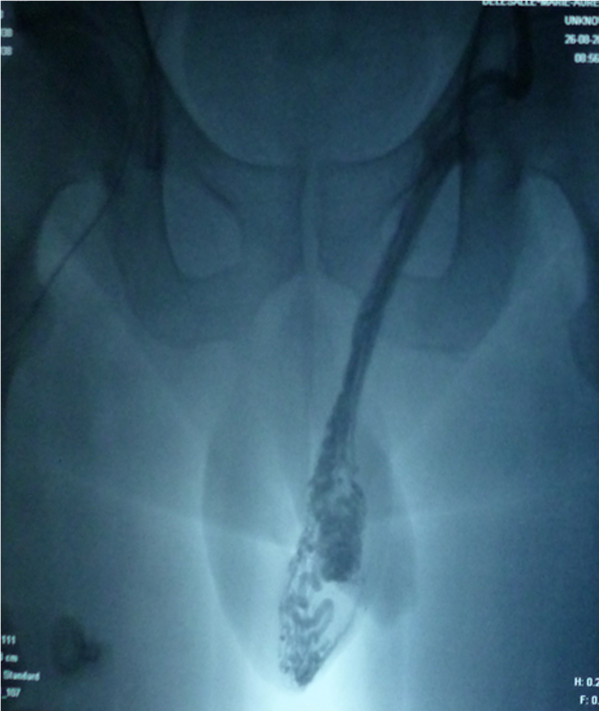
**A retrograde venogram of the testicular vein before embolization.**

**Figure 2:**
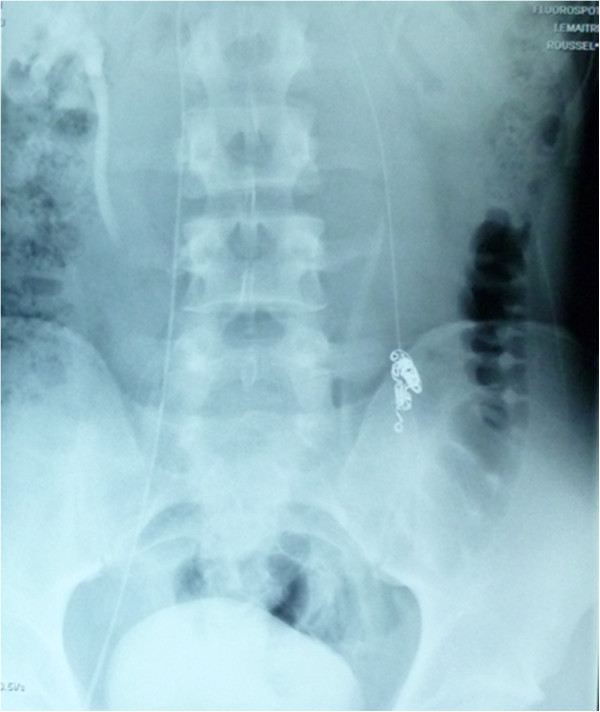
**An X-ray showing embolization coils occluding the testicular vein.**

**Figure 3:**
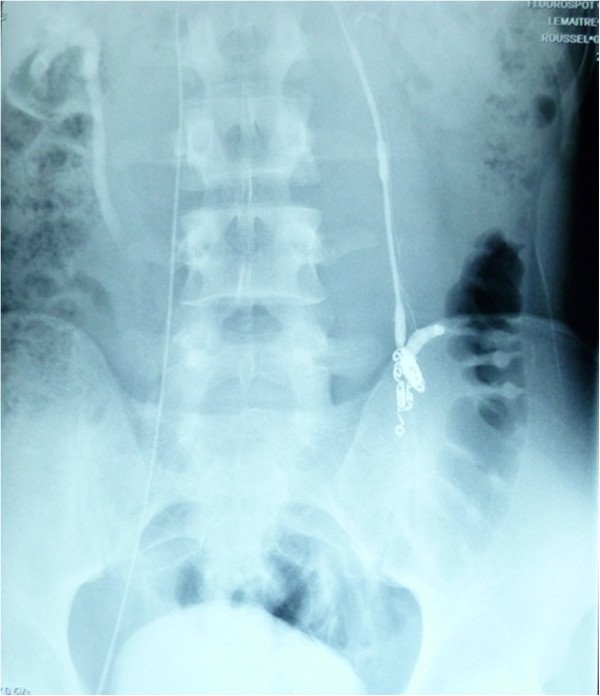
**A post-embolization venogram showing the retention of contrast medium upstream of the coils.**

All procedures were performed by the same radiologist (LL). In a prior consultation, the radiologist confirmed the indication for embolization and provided the patient with information about the procedure. Embolization was performed with the retrograde approach. The patient lay supine on a movable table (Axiom M, Siemens, Munich, Germany). After induction of local anaesthesia (xylocaine, 200 mg/20 mL), the right femoral vein was punctured with an 18G needle. Under fluoroscopic guidance, a 5 F introducer sheath (Desilet 4 F Radifocus, Terumo, Tokyo, Japan) was placed in the access site to avoid injury of the vein. A hydrophilic guide (Wire M, Terumo, Leuven, Belgium) and a 4 F angiographic catheter (C2 4 F 60 cm 38”, Cordis, Hialeah FL, USA) were then advanced to gain access to the internal spermatic vein via the inferior vena cava and the left renal vein. While the patient was in a semi-upright position, contrast medium was injected manually in order to acquire a retrograde venogram of the testicular vein as far down as the pampiniform plexus (Figure 1). We used Tornado® embolization coils (Cook Medical, Bloomington, IN, USA) (Figure 2) or AMPLATZER™ plugs (St Jude Medical, Saint Paul, MN, USA) to occlude the testicular vein and any collateral venous channels responsible for reflux. A venogram of the left iliac vein was acquired after embolization of the left testicular vein, in order to check for successful embolization and the lack of aberrant drainage pathways (Figure 3). Successful embolization was defined as the absence of contrast medium above the inserted coil or plug.

### Data analyses

All statistical analyses were performed with SAS software (version 9.2, SAS Institute Inc., Cary, NC, USA). Depending on the data distribution, the results were expressed as the mean ± standard deviation (SD) or the median [interquartile range]. The normality of the data distribution was tested in a Shapiro-Wilk test. Longitudinal data were evaluated with an analysis of variance for repeated measures. Comparisons at each time point were performed using *post hoc* tests with a Bonferroni correction. The threshold for statistical significance (notably when defining a significant pre- vs. post- embolization improvement in sperm parameters) was set to *p* < 0.05.

## Results

During the study period, 119 patients with varicocele were referred to our andrology clinic for the assessment of fertility problems. A total of forty seven infertile patients (mean ± SD age: 30.43 ± 5.16; mean duration of infertility: 33.76 ± 19.72 months) underwent embolization for grade III varicocele of the left testis and were thus included in the study. The mean left testicular volume was 12.67 ± 4.21 mL and the mean total (right plus left) testicular volume was 28.69 ± 9.49 mL. All 47 embolization procedures were technically successful, as confirmed by a venogram showing the absence of contrast medium above the inserted coil or plug. At M0, the mean serum testosterone, FSH and inhibin B levels were 4.61 ± 1.38 ng/mL, 8.91 ± 5.94 IU/L and 122.7 ± 83.84 pg/mL, respectively (Table 1). There were no significant changes over time (i.e. from M0 to M3 and then M6) in any of these levels.

**Table 1:** **Semen parameters and hormone levels before embolization (M0) and 3 and 6 months afterwards (M3 and M6, respectively)**

n=47 patients	M0	M3	M6	***p*** value
Testosterone ng/mL	4.61 ± 1.38 (4.42) [3.65-5.55]	4.49 ± 1.43 (4.14) [3.38-5.52]	4.92 ± 2.17 (4.64) [3.54-5.50]	1^a^ 0.58^b^ 0.08^c^
FSH IU/L	8.91 ± 5.94 (7.65) [4.35-11.95]	8.40 ± 4.99 (7.60) [4.40-11.50]	8.11 ± 4.88 (7.30) [4.60-10.70]	0.24^a^ 0.18^b^ 1^c^
Inhibin B pg/mL	122.7 ± 83.84 (107) [69-170]	131 ± 86.99 (111) [64.5-170]	131.7 ± 79.86 (113.5) [84-171]	1^a^ 1^b^ 1^c^
Spermatozoa 10^6^/ejaculate	28.44 ± 55.99 (5.78) [0.84-37.70]	52.59 ± 55.51 (38.75) [3.96-95]	55.19 ± 70.53 (21.75) [4.41-85.05]	<0.0003^a^ 0.0036^b^ 1^c^
% progressive motility	21.83 ± 16.48 (18.5) [8-35]	29.32 ± 14.21 (30) [20-37.50]	29.19 ± 17.84 (25) [15-40]	0.0086^a^ 0.0093^b^ 1^c^
% vitality	61.88 ± 15.98 (65.5) [52-74]	69.14 ± 14.86 (70) [61.50-80] ^2^	67.05 ± 17.10 (72) [60-80] ^2^	0.02^a^ 0.18^b^ 1^c^
% typical spermatozoa	12.88 ± 7.15 (12) [8-18.50]	19.03 ± 11.02 (16) [10-28]	19.11 ± 10.74 (17) [10-26]	0.0001^a^ 0.0002^b^ 1^c^
% thin head	14.03 ± 13.09 (10) [5-16]	9.26 ± 8.69 (7) [3-13]	6.35 ± 5.29 (4) [4-7]	0.05^a^ 0.01^b^ 1^c^
Multiple Anomalies Index	1.88 ± 0.19 (1.87) [1.75-1.98]	1.79 ± 0.23 (1.79) [1.63-1.90]	1.78 ± 0.27 (1.69) [1.59-1.96]	0.04^a^ 0.24^b^ 1^c^

Values are the mean ± SD (median) [1^st^ quartile- 3^rd^ quartile]. ^a^M0 vs. M3; ^b^M0 vs. M6; ^c^M3 vs. M6.

A sperm concentration below 39×10^6^ spermatozoa per ejaculate was observed in 36 of the 47 patients (77%). Asthenozoospermia (progressive motility a + b less than 32%) was observed in 31 of the 47 patients (65%). Twenty-two of the 47 patients (46%) had fewer than 15% of typical sperm cells.

The median sperm concentration per ejaculate at M3 (38.75 [3.96-95] × 10^6^/ejaculate) was significantly higher (p < 0.05) than the value at M0 (5.78 [0.84-37.70] × 10^6^/ejaculate). Similarly, the mean sperm progressive motility at M3 (29.32 ± 14.21%) was significantly greater value at M0 (21.83 ± 16.48%). This was also the case of mean vitality (69.14 ± 14.86% at M3, up from 61.88 ± 15.98% at M0). There were no significant changes in sperm concentration, progressive motility or vitality when comparing M3 with M6.

The mean percentage of normal sperm at M3 (19.03 ± 11.02%) was significantly greater than the value at M0 (12.88 ± 7.15%). The mean percentage of sperm with an abnormally thin head was significantly lower at M6 (6.35 ± 5.29%) than at M0 (14.03 ± 13.09%). The mean multiple anomalies index at M3 (1.79 ± 0.23) was significantly lower than at M0 (1.88 ± 0.19) decreased to. There were no significant changes in sperm morphology, thin head anomalies or the multiple anomalies index when comparing M3 with M6.

## Discussion

Men from infertile couples should be appropriately counselled on the likelihood of attaining a significant improvement in semen parameters after varicocele repair. Divergent results have been obtained when the efficacy criterion is an improvement in sperm morphology. The effect of varicocele repair on sperm head and flagella parameters is less clear. Some researchers have reported a significant increase in the percentage of spermatozoa with a normal morphology [6, 18, 19]. In contrast, Gazzera et al. [7] did not observe any significant differences in sperm morphology. The initial (and now dated) study by Czyglik et al. [20] showed an increase in the proportion of spermatozoa with an abnormally thin head. Osawa et al. [21] showed that low ICSI fertilisation rates have also been found in men with severely elongated spermatozoa compared to other morphology abnormalities. Here, we provide evidence to show that embolization is associated with a significant decrease in the proportion of spermatozoa with an abnormally thin head.

In an analysis of 9038 men at 34 centers in 24 countries, the World Health Organisation study showed that varicocele is clearly associated with infertility and impaired testicular function [22]. Although this topic has been extensively investigated since then, a variety of conclusions were reached because the physiopathology of varicocele is not completely understood. Hypotheses concerning the harmful effects of varicocele [23] include scrotal/testicular hyperthermia, elevations in serum gonadotropin levels, increased venous pressures, the accumulation of toxins, hypoxia, and hormonal imbalance. The effects of the correction of varicocele on sperm quality and reproductive potential are less well understood. Clinical research has demonstrated improved semen parameters, higher DNA integrity, and better assisted reproductive technology outcomes after varicocele repair [24].

Treatment options for varicocele can be divided into two major categories [25, 26]: (i) percutaneous occlusion via intravenous injection of various materials (embolization or sclerotherapy) and (ii) surgical ligation or clipping to prevent venous reflux, e.g. open surgical varicocelectomy (inguinal, subinguinal or retroperitoneal approaches), laparoscopic varicocelectomy and microsurgical (inguinal or subinguinal) varicocelectomy. Embolization presents several advantages: ambulatory management, the requirement for local anaesthesia only, low cost, a low failure rate (for experienced radiologists) and a low complication rate. Dewire et al. [27] showed that percutaneous embolization is equally effective in improving male fertility and costs about the same as the surgical ligature of varicocele.

Varicocele in infertile men is generally associated with Leydig cell dysfunction and hypogonadism. It has been shown that varicocelectomy significantly increases serum testosterone levels in men with hypogonadism and infertility [28, 29]. In the present study, spermatogenesis was not strongly altered and our patients did not have severe oligoasthenoteratozoospermia. We did not observe significant changes over time in serum testosterone, FSH and inhibin B concentrations. Inhibin B is an important factor in testicular hormonal function. In men with severe oligoasthenoteratozoospermia, treatment of varicocele was associated with a significant increase in inhibin B levels and a significant decrease in FSH levels [8]. Some researchers have noted a significant improvement in serum inhibin B level after varicocelectomy and have concluded that combining this endocrine marker with a spermogram could be an effective guide to the effect of varicocelectomy on spermatogenesis [12, 30]. In contrast, our present results confirmed those of Fujisawa et al. [11], in which the preoperative serum inhibin B concentration did not reliably predict the response to varicocelectomy. This discrepancy may be due to the fact that the inhibin B level was in the normal range in our study population, which suggesting that there were no major Sertoli cell disorders. This hypothesis fits with the relatively normal FSH levels observed here. In the present study, the biological presentation of men with varicocele was heterogeneous. The results of the World Health Organisation study [22] showed that clinically confirmed left varicocele was associated with a relatively small left testis. Many other studies have evidenced a relationship between testicular volume and varicocele in infertile patients [31, 32]. Our patients displayed scrotal hypotrophy but were not hypogonadal according to clinical biochemistry parameters. Furthermore, we did not observe significant changes in hormonal parameters after embolization.

## Conclusions

The present study results show that varicocele embolization (a well-tolerated technique with a high benefit-cost ratio) in men with grade III left varicocele, abnormal sperm parameters and documented infertility in the couple is associated with a significant improvement in sperm concentration, motility, vitality and morphology but not in serum testosterone, FSH and inhibin B levels. This finding supports suggestions that varicocele impairs spermatogenesis but not the testicles’ steroidogenic function. The present study was limited by the absence of data on pregnancy rates. More detailed studies are therefore required before firm conclusions on the prognosis for fertility can be drawn.
